# Supplementary material for: The brain imaging data structure, a format for organizing and describing outputs of neuroimaging experiments
Source: Sci Data. 2016 Jun 21;3:160044. doi: 10.1038/sdata.2016.44 (PMC4978148; doi:10.1038/sdata.2016.44)
Supplement: Supplementary File 1 [file sdata201644-s1.pdf]

The Brain Imaging Data Structure (BIDS) Specification

version 1.0.0-rc4

Browse example datasets:
https://github.com/INCF/BIDS-examples
Download example datasets:
https://drive.google.com/folderview?id=0B2JWN60ZLkgkMGlUY3B4MXZIZW8&usp=sharing

1 Changelog

1.1 rc4

- Replaced links to neurolex with explicit DICOM Tags.
- Added `sourcedata.`
- Added data dictionaries.
- Be more explicit about contents of JSON files for structural (anatomical) scans.

1.2 rc3

- Renamed `PhaseEncodingDirection` values from “x”, “y”, “z” to “i”, “j”, “k” to avoid confusion with FSL parameters
- Renamed `SliceEncodingDirection` values from “x”, “y”, “z” to “i”, “j”, “k”

1.3 rc2

- Removed the requirement that TSV files cannot include more than two consecutive spaces.
- Refactor of the definitions sections (copied from the manuscript)
- Make support for uncompressed .nii files more explicit.
- Added `BIDSVersion` to dataset.json
- Remove the statement that `SliceEncodingDirection` is necessary for slice time correction
- Change dicom converter recommendation from dcmstack to dcm2nii and dicm2nii following interactions with the community (see https://github.com/moloney/dcmstack/issues/39 and https://github.com/neurolabusc/dcm2niix/issues/4)
- Added section on behavioral experiments with no accompanying MRI acquisition
- Add `_magnitude.nii[.gz]` image for GE type fieldmaps.
- Replaced `EchoTimeDifference` with `EchoTime1` and `EchoTime2` (SPM toolbox requires this input).
- Added support for single band reference image for DWI.
- Added `DatasetDOI` field in the dataset description.
- Added description of more metadata fields relevant to DWI fieldmap correction.
- `PhaseEncodingDirection` is now expressed in “x”, “y” etc. instead of “PA” “RL” for DWI scans (so it’s the same as BOLD scans)
- Added `rec-<label>` flag to BOLD files to distinguish between different reconstruction algorithms (analogous to anatomical scans).

- Added recommendation to use `_physio` suffix for continuous recordings of motion parameters obtained by the scanner side reconstruction algorithms.

1.4 rc1

- Initial release

2 Table of contents

1 Changelog
    1.1 rc4
    1.2 rc3
    1.3 rc2
    1.4 rc1
2 Table of contents
3 Introduction
    3.1 Motivation
    3.2 Definitions
    3.3 Compulsory, optional, and additional
    3.4 Raw vs. derived data
    3.5 The Inheritance Principle
    3.6 Extensions
4 File Format specification
    4.1 Imaging files
    4.2 Tabular files
        4.2.1 Example:
        4.2.2 Example:
    4.3 Key/value files (dictionaries)
        4.3.1 Example:
5 Subject naming
6 Units
7 Directory structure (single session example)
8 Detailed file descriptions
    8.1 Dataset description
    8.2 Code
    8.3 Anatomy imaging data
    8.4 Task (including resting state) imaging data
        8.4.1 Example:
    8.5 Task events
        8.5.1 Example:
        8.5.1.1 Example:
    8.6 Physiological and other continuous recordings
        8.6.1 Example:
    8.7 Behavioral experiments (with no MRI)

8.8 Diffusion imaging data
8.8.1 bvec example:
8.8.2 bval example:
8.8.3 json example:
8.9 Fieldmap data
8.9.1 Case 1: Phase difference image and at least one magnitude image
8.9.2 Case 2: Two phase images and two magnitude images
8.9.3 Case 3: A single, real fieldmap image (showing the field inhomogeneity in each voxel)
8.9.4 Case 4: Multiple phase encoded directions (topup)
8.10 Scans key file
8.10.1 Example:
8.11 Participant key file
8.11.1 Single session example:
9 Longitudinal studies with multiple sessions (visits)
9.1 Sessions file
9.1.1 Multiple sessions example:
12 Appendix I: Licenses
12.1 What is below is not part of the specifications : brainstorming / material / idea ...
12.1.1 Proposal: Switch from JSON to YAML
12.1.2 Question:
12.1.3 IDEA: BIDS also for internal long-term data storage (Enrico Glerean)

3 Introduction

3.1 Motivation

Neuroimaging experiments result in complicated data that can be arranged in many different ways. So far there is no consensus how to organize and share data obtained in neuroimaging experiments. Even two researchers working in the same lab can opt to arrange their data in a different way. Lack of consensus (or a standard) leads to misunderstandings and time wasted on rearranging data or rewriting scripts expecting certain structure. Here we describe a simple and easy to adopt way of organising neuroimaging and behavioural data. By using this standard you will benefit in the following ways:

- It will be easy for another researcher to work on your data. To understand the organisation of the files and their format you will only need to refer them to this document. This is especially important if you are running your own lab and anticipate more than one person working on the same data over time. By using BIDS you will save time trying to understand and reuse data acquired by a graduate student or postdoc that has already left the lab.
- There is a growing number of data analysis software packages that can understand data organised according to BIDS.
- Databases such as OpenfMRI.org accept datasets organised according to BIDS. If you ever plan to share your data publicly (nowadays some journals require this) you can minimize the additional time and energy spent on publication, and speed up the curation process by using BIDS to structure and describe your data right after acquisition.

- There are validation tools that can check your dataset integrity and let you easily spot missing values.

BIDS is heavily inspired by the format used internally by OpenfMRI.org. While working on BIDS we consulted many neuroscientists to make sure it covers most common experiments, but at the same time is intuitive and easy to adopt. The specification is intentionally based simple file formats and folder structures to reflect current lab practices and make it accessible to wide range of scientists coming from different backgrounds.

3.2 Definitions

Throughout this protocol we use a list of different MRI related terms. To avoid misunderstanding we clarify them here.

1. **Dataset** - a set of neuroimaging and behavioural data acquired for a purpose of a particular study. A dataset consists of data acquired from one or more subjects, possibly from multiple sessions.
2. **Subject** - a person or animal participating in the study.
3. **Session** - a logical grouping of neuroimaging and behavioural data consistent across subjects. Session can (but doesn't have to) be synonymous to a visit in a longitudinal study. In general, subjects will stay in the scanner during one session. However if a subject has to leave the scanner room and then be re-positioned on the scanner bed, the set of MRI acquisitions will still be considered as a session and match sessions acquired in other subjects. Similarly in situations where different type of data is obtained over several visits (for example fMRI on one day followed by DWI the day after) those can be grouped in one session. Defining multiple sessions is appropriate when several identical or similar data acquisitions are planned and performed on all -or most- subjects, often in the case of some intervention between sessions (e.g., training).
4. **MRI acquisition** - a continuous uninterrupted block of time during which an MRI scanner was acquiring data according to particular scanning sequence/protocol.
5. **Data type** - a functional group of different types of MRI data. In BIDS we define four data types: `func` (task based and resting state functional MRI), `dwi` (diffusion weighted imaging), `fmap` (field inhomogeneity mapping data such as field maps), `anat` (structural imaging such as T1, T2, etc.)
6. **Task** - a set of activities performed by the subject during MRI acquisition (usually BOLD sensitive functional MRI) that aim at eliciting changes in the brain. Tasks are usually accompanied by stimuli and responses, and can greatly vary in complexity. For the purpose of this protocol we consider the so-called “resting state” a task. For simplicity, a task is always tied to one MRI acquisition. Therefore, even if during one acquisition the subject performed multiple conceptually different behaviours (with different sets of instructions) they will be considered one (combined) task.
7. **Event** - a stimulus or subject response recorded during a task. Each event has an onset time and duration. Note that not all tasks will have recorded events (e.g., resting state).
8. **Run** - an uninterrupted repetition of an MRI acquisition that has the same acquisition parameters and task (however events can change from run to run due to different subject response or randomized nature of the stimuli).

3.3 Compulsory, optional, and additional

The following standard describes a way of arranging data and writing down metadata for a subset of neuroimaging experiments. Some aspects of the standard are compulsory. For example a particular file name

format is required when saving structural scans. Some aspects are regulated but optional. For example a T2 volume does not need to be included, but when it is available is should be saved under a particular file name specified in the standard.
This standard aspires to describe a majority of datasets, but acknowledges that there will be cases that do not fit. In such cases one can include additional files and subfolders to the existing folder structure following common sense. For example one may want to include eye tracking data in a vendor specific format that is not covered by this standard. The most sensible place to put it is next to the continuous recording file with the same naming scheme but different extensions. The solutions will change from case to case and publicly available datasets will be reviewed to include common data types in the future releases of the BIDS spec.

3.4 Raw vs. derived data

BIDS in its current form is designed to normalize and describe raw (unprocessed or minimally process due to file format conversion) data. During analysis such data will be transformed and partial as well as final results will be saved. Derivatives of the raw data (other than products of DICOM to NIftI conversion) should be kept separate from the raw data. This way one can protect the raw data from accidental changes by file permissions. In addition it is easy to distinguish partial results from the raw data and share the latter. This specification does not go into details of recommending a particular naming scheme for including different types of source data (raw event logs, parameter files, etc.) and data derivatives (correlation maps, brain masks, contrasts maps, etc.). However, in the case that these data are to be included:

1. We do recommend keeping them in separate "`sourcedata`" and "`derivatives`" folders each with a similar folder structure as presented below for the BIDS-managed data. For example: `derivatives/sub-01/ses-pre/mask.nii.gz` or `sourcedata/sub-01/ses-pre/MyEvent.sce`.
2. A `README` file should be found at the root of the "`sourcedata`" or the "`derivatives`" folder (or both). This file should describe the nature of the raw data or the derived data. In the case of the existence of a "`derivatives`" folder, we recommend to include details about the software stack and settings used to generate the results. Non-imaging objects to improve reproducibility are encouraged (scripts, setting files, etc.).
3. We encourage to include the pdf print-out with the actual sequence parameters generated by the scanner in the "`sourcedata`" folder.

3.5 The Inheritance Principle

Any metadata file (.json, .bvec, .tsv, etc.) may be defined at any directory level. The values from the top level are inherited by all lower levels unless they are overridden by a file at the lower level. For example, tasks.tsv may be specified at the participant level, setting TR to a specific value. If one of the series has a different TR than the one specified in that file, another tasks.tsv file can be placed within that specific series directory specifying the TR for that specific run.
There is no notion of "unsetting" a key/value. For example if there is a JSON file corresponding to particular subject/run defining a key/value and there is a JSON file on the root level of the dataset that does not define this key/value it will not be "unset" for all subjects/runs.

3.6 Extensions

BIDS specification can be extended. Currently we are working on Model and hypothesis specification is one such extension.

4 File Format specification

4.1 Imaging files

Imaging data are stored using the NIfTI file format, preferably compressed NIfTI files (.nii.gz), either 1.0 or 2.0 version. Imaging data should be converted to the NIfTI format using a tool that provides as much of the NIfTI header information (such as orientation and slice timing information) as possible. Since the NIfTI standard offers limited support for the various image acquisition parameters available in DICOM files, we also encourage users to provide additional meta information extracted from DICOM files in a sidecar JSON file (with the same filename as the .nii[.gz] file, but with a .json extension). Extraction of BIDS compatible metadata can be performed using dcm2nii (https://www.nitrc.org/projects/dcm2nii/) and dicm2nii (http://www.mathworks.com/matlabcentral/fileexchange/42997-dicom-to-nifti-converter/content/dicm2nii.m) DICOM to NIfTI converters. A provided validator (https://github.com/INCF/bids-validator) will check for conflicts between the JSON file and the data recorded in the NIfTI header.

4.2 Tabular files

Other files are saved as tab delimited values (.tsv) files, aka csv files where commas are replaced by tabs. Tabs need to be true tab characters and not a series of space characters. Each TSV file needs to start with a header line listing the names of all columns (with the exception for physiological and other continuous acquisition data - see below for details). Names need to be separated with tabs. String values containing tabs must be escaped using double quotes.
Missing values should be coded as “n/a”.

4.2.1 Example:[1]

```
onset duration    response_time     correct     stop_trial  go_trial
200   20          0                 n/a         n/a         n/a
```

Tabular files can be optionally accompanied by a simple data dictionary in a JSON format (see below). The data dictionaries should have the same name as their corresponding tabular files but with .json extensions. Each entry in the data dictionary has a name corresponding to a column name and the following fields:

- LongName - Long (unabbreviated) name of field.
- Description - Description of the field.
- Levels - For categorical variables: a dictionary of possible values (keys) and their descriptions (values).
- Units - Measurement units.
- TermURL - URL pointing to a formal definition in an ontology available on the web.

[1] Note that to make the display clearer, the second row does contain two successive tabs, which should not happen in an actual BIDS tsv file.

4.2.2 Example:

```
{
  "education": {
    "LongName": "Education level",
    "Description": "Education level, self-rated by participant",
    "Levels": {
      "1": "Finished primary school",
      "2": "Finished secondary school",
      "3": "Student at university",
      "4": "Has degree from university"
    }
  },
  "bmi": {
    "LongName": "Body mass index",
    "Units": "kilograms per squared meters",
    "TermURL": "http://purl.bioontology.org/ontology/SNOMEDCT/60621009"
  }
}
```

4.3 Key/value files (dictionaries)

Javascript Object Notation Style (JSON) files will be used for storing key/value pairs. Extensive documentation of the format can be found here: http://json.org/. Several editors have built-in support for JSON syntax highlighting that aids manual creation of such files. An online editor for JSON with built-in validation is available at: http://jsoneditoronline.org. JSON files should be in UTF-8 encoding.

4.3.1 Example:

```
{
    "RepetitionTime": 3.0,
    "Instruction": "Lie still and keep your eyes open"
}
```

5 Subject naming

Subjects (participants) should be assigned unique labels. Labels can consist of letters and/or numbers. If numbers are used we strongly encourage zero padding ("01" instead of "1" if you have more than nine subjects) to make alphabetical sorting more intuitive.

6 Units

Elapsed time should be expressed in seconds. Please note that some DICOM parameters have been traditionally expressed in milliseconds. Those need to be converted to seconds.

Frequency should be expressed in Hertz.

Describing dates and timestamps:

- Date time information should be expressed in the following format YYYY-MM-DDThh:mm:ss (ISO8601 date-time format). For example: 2009-06-15T13:45:30
- Time stamp information should be expressed in the following format: 13:45:30
- Dates can be shifted by a random number of days for privacy protection reasons. To distinguish real dates from shifted dates always use year 1900 or earlier when including shifted years. For longitudinal studies please remember to shift dates within one subject by the same number of days to maintain the interval information. Example: 1867-06-15T13:45:30
- Age should be given as the number of years since birth at the time of scanning (or first scan in case of multi session datasets). Using higher accuracy (weeks) should be avoided due to privacy protection.

7 Directory structure (single session example)

This is an overview of the folder and file structure. Because there is only one session, the session level is not required by the format. For details on individual files see descriptions in the next section:

- **`sub-control01`**
  - **`anat`**
    - `sub-control01_T1w.nii.gz`
    - `sub-control01_T1w.json`
    - `sub-control01_T2w.nii.gz`
    - `sub-control01_T2w.json`
  - **`func`**
    - `sub-control01_task-nback_bold.nii.gz`
    - `sub-control01_task-nback_bold.json`
    - `sub-control01_task-nback_events.tsv`
    - `sub-control01_task-nback_physio.tsv.gz`
    - `sub-control01_task-nback_physio.json`
    - `sub-control01_task-nback_sbref.nii.gz`
  - **`dwi`**
    - `sub-control01_dwi.nii.gz`
    - `sub-control01_dwi.bval`
    - `sub-control01_dwi.bvec`
  - **`fmap`**
    - `sub-control01_phasediff.nii.gz`
    - `sub-control01_phasediff.json`
    - `sub-control01_magnitude1.nii.gz`
  - `sub-control01_scans.tsv`
  - Additional files and folders containing raw data may be added as needed for special cases. They should be named using all lowercase with a name that reflects the nature of the scan (e.g., “calibration”). Naming of files within the directory should follow the same scheme as above (e.g., “sub-control01_calibration_Xcalibration.nii.gz”)
- **`code`**

- deface.py
- **derivatives**
- participants.tsv
- dataset_description.json
- README
- CHANGES

8 Detailed file descriptions

8.1 Dataset description

Template: dataset_description.json, README, and CHANGES

A JSON file describing the dataset. Every dataset need to include this file with the following mandatory fields:

- Name: name of the dataset
- BIDSVersion: The version of the BIDS standard that was used

In addition the following fields can be provided:

- License: what license is this dataset distributed under? The use of license name abbreviations is suggested for specifying a license. A list of common licenses with suggested abbreviations can be found in appendix III.
- Authors: List of individuals who contributed to the creation/curation of the dataset
- Acknowledgements: who should be acknowledge in helping to collect the data
- HowToAcknowledge: Instructions how researchers using this dataset should acknowledge the original authors. This field can also be used to define a publication that should be cited in publications that use the dataset,
- Funding: sources of funding (grant numbers)
- ReferencesAndLinks: a list of references to publication that contain information on the dataset, or links.
- DatasetDOI: the Document Object Identifier of the dataset (not the corresponding paper).

Example:

```
{
    "Name": "The mother of all experiments",
    "BIDSVersion":  "1.0.0rc1",
    "License": "CC0",
    "Authors": ["Ramon y Cajal"],
    "Acknowledgements": "say here what are your acknowledgments",
    "HowToAcknowledge": "say here how you would like to be acknowledged",
    "Funding": "list your funding sources",
    "ReferencesAndLinks": ["a data paper", "a resource to be cited when using
the data"],
    "DatasetDOI": "10.0.2.3/dfjj.10"
```

```
}
```

In addition a free form text file (`README`) describing the dataset in more details should be provided. Version history of the dataset (describing changes, updates and corrections) can be provided in a form of a `CHANGES` text file. This file should follow the following the CPAN Changelog convention: http://search.cpan.org/~haarg/CPAN-Changes-0.400002/lib/CPAN/Changes/Spec.pod. `README` and `CHANGES` files should be either i ASCII or UTF-8 encoding .

Example:

```
1.0.1 2015-08-27

  - Fixed slice timing information.

1.0.0 2015-08-17

  - Initial release.
```

8.2 Code

Template:

```
code/*
```

Source code of scripts that were used to prepare the dataset (for example if it was anonymized or defaced) can be stored here.[2] Extra care should be taken to avoid including original IDs or any identifiable information with the source code. There are no limitations or recommendation on the language and or code organization of these scripts at the moment.

8.3 Anatomy imaging data

Template:

```
sub-<participant_label>/
      anat/
            sub-<participant_label>[_acq-<label>][_rec-<label>][_run-<index>]_T2w.nii[.gz]
```

Anatomical (structural) data acquired for that participant. Possible modalities include, but are not limited to:

Name	Suffix	Description
T1 weighted	`_T1w`	
T2 weighted	`_T2w`	
T1 map	`_T1map`	quantitative T1 map (likewise for T2)

[2] Storing actual source files with the data is preferred over links to external source repositories to maximize long term preservation (which would suffer if an external repository would not be available anymore).

T2 map	_T2map	quantitative T2 map
FLAIR	_FLAIR	
Proton density	_PD	
Combined PD/T2	_PDT2	
Inplane T1	_inplaneT1	T1-weighted anatomical image matched to functional acquisition
Inplane T2	_inplaneT2	T2-weighted anatomical image matched to functional acquisition
Angiography	_angio	
Defacing mask	_defacemask	Mask used for defacing
Susceptibility Weighted Imaging (SWI)	_SWImagandphase	Magnitude and corresponding phase images of the SWI

Several scans of the same modality can be acquired and will be indexed with a suffix: _run-01, _run-02, _run-03 etc. When there is only one scan of a given type the suffix can be omitted. Please note that diffusion imaging data is stored elsewhere (see below). Metadata corresponding to each anatomical NifTi file can be provided in a JSON file with the same name but .json extension.
The optional "acq-<label>" key/value pair corresponds to a custom label the user may use to distinguish a different set of parameters used for acquiring the same modality. For example this should be used when a study includes two T1w images - one full brain low resolution and and one restricted field of view but high resolution. In such case two files could have the following names: sub-01_acq-highres_T1w.nii.gz and sub-01_acq-lowres_T1w.nii.gz, however the user is free to choose any other label than "highres" and "lowres" as long as they are consistent across subjects and sessions.
Similarly the optional "rec-<label>" key/value can be used to distinguish different reconstruction algorithms (for example ones using motion correction).

Some meta information about the acquisition can be provided in an additional JSON file. See **Task (including resting state) imaging data** for a list of terms and their definitions.

8.4 Task (including resting state) imaging data

Template:

```
sub-<participant_label>/
        func/
             sub-<participant_label>_task-<task_label>[_acq-<label>][_rec-<label>][_run-<index>]_bold.nii[.gz]
```

Imaging data acquired during BOLD imaging. This includes but is not limited to task based fMRI **as well as resting state fMRI (i.e. rest is treated as another task)**. For task based fMRI a corresponding task events file (see below) must be provided (please note that this file is not necessary for resting state scans). For multiband

acquisitions, one can also save the single-band reference image as type "sbref" (e.g. `sub-control01_task-nback_sbref.nii.gz`).

Each task has a unique label consisting of letters and/or numbers (other characters including spaces and underscores are not allowed). Those labels have to be consistent across subjects and sessions.
If more than one run of the same task has been acquired a key/value pair: `_run-01`, `_run-02`, `_run-03` etc. can be used. If only one run was acquired the suffix can be omitted. In the context of functional imaging a run is defined as the same task, but a different set of stimuli (for example randomized order) and participant responses.
The optional "`acq-<label>`" key/value pair correspond to a custom label user may use to distinguish different set of parameters used for acquiring the same task. For example this should be used when a study includes two resting state images - one single band and one multiband. In such case two files could have the following names: `sub-01_task-rest_acq-singleband_bold.nii.gz` and `sub-01_task-rest_acq-multiband_bold.nii.gz`, however the user is free to choose any other label than "singleband" and "multiband" as long as they are consistent across subjects and sessions.
Similarly the optional "`rec-<label>`" key/value can be used to distinguish different reconstruction algorithms (for example ones using motion correction).

Some meta information about the acquisition needs to be provided in an additional JSON file.
Required fields:

- `RepetitionTime`: The time in seconds between the beginning of an acquisition of one volume and the beginning of acquisition of the volume following it (TR). Please note that this definition includes time between scans (when no data has been acquired) in case of sparse acquisition schemes. This value needs to be consistent with the '`pixdim[4]`' field (after accounting for units stored in '`xyzt_units`' field) in the NIFTI header.
- `TaskName`: Name of the task (for resting state use the "rest" prefix). No two tasks should have the same name. Task label is derived from this field by removing all non alphanumeric ([a-zA-Z0-9]) characters.

Other recommended metadata.
MRI acquisition parameters are divided into several categories based on ["A checklist for fMRI acquisition methods reporting in the literature"] by Ben Inglis:

- Scanner Hardware
  - `Manufacturer`: Manufacturer of the equipment that produced the composite instances. Corresponds to DICOM Tag 0008, 0070 "Manufacturer"
  - `ManufacturersModelName`: Manufacturers model name of the equipment that produced the composite instances. Corresponds to DICOM Tag 0008, 1090 "Manufacturers Model Name"
  - `MagneticFieldStrength`: Nominal field strength of MR magnet in Tesla. Corresponds to DICOM Tag 0018,0087 "Magnetic Field Strength"
  - `HardcopyDeviceSoftwareVersion`: Manufacturer's designation of the software of the device that created this Hardcopy Image. Corresponds to DICOM Tag 0018, 101A "Hardcopy Device Software Version"

- `ReceiveCoilName`: Information describing the receiver coil (Note: This isn't a consistent field name across vendors. This name is the dcmstack output from a GE dataset, but it doesn't seem to be simply coded into the dcmstack output for a Siemens scan). This doesn't correspond to a term in the DICOM ontology
- `GradientSetType`: It should be possible to infer the gradient coil from the scanner model. If not,*e.g.* because of a custom upgrade or use of a gradient insert set, then the specifications of the actual gradient coil should be reported independently.
- `MRTransmitCoilSequence`: This is a relevant field if a non-standard transmit coil is used. Corresponds to DICOM Tag 0018, 9049 "MR Transmit Coil Sequence"
- `MatrixCoilMode`: (If used) A method for reducing the number of independent channels by combining in analog the signals from multiple coil elements. There are typically different default modes when using un-accelerated or accelerated (*e.g.* GRAPPA, SENSE) imaging.
- `CoilCombinationMethod`: Almost all fMRI studies using phased-array coils use root-sum-of-squares (rSOS) combination, but other methods exist. The image reconstruction is changed by the coil combination method (as for the matrix coil mode above), so anything non-standard should be reported.

- In-Plane Spatial Encoding
  - `PulseSequenceType`: A general description of the pulse sequence used for the scan (I.e. MPRAGE, Gradient Echo EPI, Spin Echo EPI, Multiband gradient echo EPI)
  - `PulseSequenceDetails`: Information beyond pulse sequence type that identifies the specific pulse sequence used (I.e. "Standard Siemens Sequence distributed with the VB17 software," "Siemens WIP ### version #.##," or "Sequence written by X using a version compiled on MM/DD/YYYY")
  - `NumberShots`: The number of RF excitations need to reconstruct a slice or volume. Please mind that this is not the same as Echo Train Length which denotes the number of lines of k-space collected after an excitation.
  - `ParallelReductionFactorInPlane`: The parallel imaging (e.g, GRAPPA) factor. Use the denominator of the fraction of k-space encoded for each slice. For example, 2 means half of k-space is encoded. Corresponds to DICOM Tag 0018, 9069 "Parallel Reduction Factor In-plane".
  - `ParallelAcquisitionTechnique`: The type of parallel imaging used (e.g. GRAPPA, SENSE). Corresponds to DICOM Tag 0018, 9078 "Parallel Acquisition Technique".
  - `PartialFourier`: The fraction of partial Fourier information collected. Corresponds to DICOM Tag 0018, 9081 "Partial Fourier".
  - `PartialFourierDirection`: The direction where only partial Fourier information was collected. Corresponds to DICOM Tag 0018, 9036 "Partial Fourier Direction".
  - `PhaseEncodingDirection`: Possible values: "i", "j", "k", "i-", "j-", "k-". The letters "i", "j", "k" correspond to the first, second and third axis of the data in the NIFTI file. The polarity of the phase encoding is assumed to go from zero index to maximum index unless '-' sign is present (then the order is reversed - starting from the highest index instead of zero). `PhaseEncodingDirection` is defined as the direction along which phase is was modulated which may result in visible distortions. Note that this is not the same as the DICOM term InPlanePhaseEncodingDirection which can have "ROW" or "COL" values. **This parameter is**

required if a corresponding fieldmap data is present or when using multiple runs with different phase encoding directions (which can be later used for field inhomogeneity correction).
    - `EffectiveEchoSpacing`: The sampling interval also known as the dwell time; required for unwarping distortions using field maps; **expressed in seconds.** See here how to calculate it. **This parameter is required if a corresponding fieldmap data is present.**
- Timing Parameters:
    - `EchoTime`: The echo time (TE) for the acquisition, specified in seconds. May be a list of values separated for multi-echo acquisitions. **This parameter is required if a corresponding fieldmap data is present.** Corresponds to DICOM Tag 0018, 0081 "Echo Time".
    - `SliceTiming`: The time at which each slice was acquired during the acquisition. Slice timing is not slice order - it describes the time (sec) of each slice acquisition in relation to the beginning of volume acquisition. It is described using a list of times (In JSON format) referring to the acquisition time for each slice. The list goes through slices along the slice axis in the slice encoding dimension (see below). **This parameter is required for sparse sequences. In addition without this parameter slice time correction will not be possible.**
    - `SliceEncodingDirection`: Possible values: "i", "j", "k", "i-", "j-", "k-" (corresponding to the first, second and third axis of the data in the NIFTI file; '-' sign corresponds to reverse order - starting from the highest index instead of zero). The axis of the NIFTI data along which a slices were acquired. This value needs to be consistent with the 'slice_dim' field in the NIFTI header.
- RF & Contrast
    - `FlipAngle`: Flip angle for the acquisition, specified in degrees. Corresponds to: DICOM Tag 0018, 1314 "Flip Angle".
- Slice Acceleration
    - `MultibandAccelerationFactor`: The multiband factor, for multiband acquisitions
- fMRI task information
    - `Instructions`: Text of the instructions given to participants before the scan. This is especially important in context of resting state fMRI and distinguishing between eyes open and eyes closed paradigms.
    - `TaskDescription`: Longer description of the task.
    - `CogAtlasID`: URL of the corresponding Cognitive Atlas Task term
    - `CogPOID`: URL of the corresponding CogPO term

When adding additional metadata please use the camelcase version of DICOM ontology terms whenever possible.

8.4.1 Example:

```
sub-control01/
      func/
            sub-control01_task-nback_bold.json
{
    "RepetitionTime": 3.0,
    "EchoTime": 0.03,
    "FlipAngle": 78,
```

```
    "SliceTiming": [0.0, 0.2, 0.4, 0.6, 0.8, 1.0, 1.2, 1.4, 1.6, 1.8, 2.0, 2.2,
2.4, 2.6, 2.8],
    "MultibandAccellerationFactor": 4,
    "ParallelReductionFactorInPlane": 2,
    "PhaseEncodingDirection": "j"
}
```

If this information is the same for all participants, sessions and runs it can be provided in `task-<task_label>_bold.json` (in the root directory of the dataset). However, if the information differs between subjects/runs it can be specified in the `sub-<participant_label>/func/sub-<participant_label>_task-<task_label>[_acq-<label>][_run-<index>]_bold.json` file. If both files are specified the one corresponding to a particular participant, task and run takes precedence.

8.5 Task events

Template:

```
sub-<participant_label>/
      func/
            <matches>_events.tsv
```

Where `<matches>` corresponds to task file name. For example: `sub-control01_task-nback`

The purpose of this file is to describe timing and other properties of events recorded during the scan. Those can be both stimuli presented to the participant, or participant responses. Each task events file requires a corresponding task imaging data file. The first two columns are mandatory and should contain "`onset`" and "`duration`" in that order. Both should be expressed in seconds (not milliseconds). Timing should be measured from the beginning of the acquisition of the first volume in the corresponding task imaging data file. Therefore negative numbers in "`onset`" are allowed[3] (however "`duration`" needs to be always positive and greater than zero). If response time was measured it should be included as the "`response_time`" column also expressed in seconds. An arbitrary number of additional columns can be added. Those allow describing other properties of events that could be later referred in modelling and hypothesis extensions of BIDS.

8.5.1 Example:

```
sub-control01/
      func/
            sub-control01_task-nback_events.tsv
onset       duration    trial_type  response_time
1.2         0.6         go          1.435
5.6         0.6         stop        1.739
```

[3] For example in case there is an in scanner training phase that begins before the scanning sequence has started events from this sequence should have negative onset time counting down to the beginning of the acquisition of the first volume.

References to existing databases can also be encoded using additional columns. Example 2 includes references to the Karolinska Directed Emotional Faces (KDEF) database[4]:

8.5.1.1 Example:

```
sub-control01/
      func/
            sub-control01_task-nback_events.tsv
onset       duration    trial_type  identifier  database    response_time
1.2         0.6         afraid      AF01AFAF    kdef        1.435
5.6         0.6         angry       AM01AFAN    kdef        1.739
5.6         0.6         sad         AF01ANSA    kdef        1.739
```

8.6 Physiological and other continuous recordings

Template:

```
sub-<participant_label>/
      func/
            <matches>[_recording-<label>]_physio.tsv.gz
```

and

```
sub-<participant_label>/
      func/
            <matches>[_recording-<label>]_physio.json

sub-<participant_label>/
      func/
            <matches>[_recording-<label>]_stim.tsv.gz
```

and

```
sub-<participant_label>/
      func/
            <matches>[_recording-<label>]_stim.json
```

Optional: yes

Where <matches> corresponds to task file name without the _bold.nii[.gz] suffix. For example: sub-control01_task-nback_run-01. If the same continuous recording has been used for all subjects (for example in the case where they all watched the same movie) one file can be used and placed in the root directory. For example: task-movie_stim.tsv.gz

Physiological recordings such as cardiac and respiratory signals and other continuous measures (such as parameters of a film or audio stimuli) can be specified using two files: a gzip compressed TSV file with data (without header line) and a JSON for storing start time, sampling frequency, and name of the columns from the TSV. Please note that in contrast to other TSV files this one does not include a header line. Instead the name of

---

[4] http://www.emotionlab.se/resources/kdef

columns are specified in the JSON file. This is to improve compatibility with existing software (FSL PNM) as well as make support for other file formats possible in the future. Start time should be expressed in seconds with relation of the time of acquisition of first volume in the series (negative values are allowed). Sampling frequency should be expressed in Hz. The following naming conventions should be used:

- `cardiac`: continuous pulse measurement
- `respiratory`: continuous breathing measurement
- `trigger`: continuous measurement of the scanner trigger signal

Any combination of those three can be included as well as any other stimuli related continuous variables (such as low level image properties in a video watching paradigm).

Physiological recordings (including eye tracking) should use the `_physio` suffix, and signals related to the stimulus should use `_stim` suffix. For motion parameters acquired from scanner side motion correction please use `_physio` suffix.

More than one continuous recording file can be included (with different sampling frequencies). In such case use different labels. For example: `_recording-contrast`, `_recording-saturation`. The full file name could then look like this: `sub-control01_task-nback_run-02_recording-movie_stim.tsv.gz`

8.6.1 Example:

```
sub-control01/
      func/
            sub-control01_task-nback_physio.tsv.gz (after decompression)
34          110            0
44          112            0
23          100            1

sub-control01/
      func/
            sub-control01_task-nback_physio.json
{
    "SamplingFrequency": 100.0,
    "StartTime": -22.3,
    "Columns": ["cardiac", "respiratory", "trigger"]
}
```

8.7 Behavioral experiments (with no MRI)

Template:

```
sub-<participant_label>/
      beh/
            task-<task_name>_events.tsv
sub-<participant_label>/
```

```
        beh/
                task-<task_name>_beh.json
sub-<participant_label>/
        beh/
                task-<task_name>_physio.tsv.gz
sub-<participant_label>/
        beh/
                task-<task_name>_physio.json
sub-<participant_label>/
        beh/
                task-<task_name>_stim.tsv.gz
sub-<participant_label>/
        beh/
                task-<task_name>_stim.json
```

In addition to logs from behavioral experiments performed along MRI acquisitions one can also include data from experiments performed outside of the scanner. The results of those experiments can be stored in the `beh` folder using the same formats for event timing (`_events.tsv`) , metadata (`_beh.json`), physiological (`_physio.tsv.gz`, `_physio.json`) and other continuous recordings (`_stim.tsv.gz`, `_stim.json`) as for tasks performed during MRI acquisitions.

8.8 Diffusion imaging data

Template:

```
sub-<participant_label>/
        dwi/
                sub-<participant_label>[_acq-<label>][_run-<index>]_dwi.nii[.gz]

sub-<participant_label>/
        dwi/
                sub-<participant_label>[_acq-<label>][_run-<index>]_dwi.bval

sub-<participant_label>/
        dwi/
                sub-<participant_label>[_acq-<label>][_run-<index>]_dwi.bvec

sub-<participant_label>/
        dwi/
                sub-<participant_label>[_acq-<label>][_run-<index>]_dwi.json
```

Diffusion-weighted imaging data acquired for that participant. The optional "`acq-<label>`" key/value pair corresponds to a custom label the user may use to distinguish different set of parameters. For example this should be used when a study includes two diffusion images - one single band and one multiband. In such case

two files could have the following names: `sub-01_acq-singleband_dwi.nii.gz` and `sub-01_acq-multiband_dwi.nii.gz`, however the user is free to choose any other label than "singleband" and "multiband" as long as they are consistent across subjects and sessions.
For multiband acquisitions, one can also save the single-band reference image as type "sbref" (e.g. `dwi/sub-control01_sbref.nii[.gz]`).
The bvec and bval files are in the FSL format[5]: The bvec files contain 3 rows with n space-delimited floating-point numbers (corresponding to the n volumes in the relevant Nifti file). The first row contains the x elements, the second row contains the y elements and third row contains the z elements of a unit vector in the direction of the applied diffusion gradient, where the i-th elements in each row correspond together to the i-th volume with [0,0,0] for non-diffusion-weighted volumes.

8.8.1 bvec example:

```
0 0 0.021828 -0.015425 -0.70918 -0.2465
0 0 0.80242 0.22098 -0.00063106 0.1043
0 0 -0.59636 0.97516 -0.70503 -0.96351
```

The bval file contains the b-values (in s/mm^2) corresponding to the volumes in the relevant Nifti file), with 0 designating non-diffusion-weighted volumes, space-delimited.

8.8.2 bval example:

```
0 0 2000 2000 1000 1000
```

.bval and .bvec files can be saved on any level of the directory structure and thus define those values for all sessions and/or subjects in one place (see Inheritance principle).

The following metadata can be included in the corresponding JSON file. All of them are optional, but their inclusion is highly recommended, because they are crucial for some data processing steps.

- `PhaseEncodingDirection`: Possible values: "i", "j", "k", "i-", "j-", "k-". The letters "i", "j", "k" correspond to the first, second and third axis of the data in the NIFTI file. The polarity of the phase encoding is assumed to go from zero index to maximum index unless '-' sign is present (then the order is reversed - starting from the highest index instead of zero). `PhaseEncodingDirection` is defined as the direction along which phase is was modulated which may result in visible distortions. Note that this is not the same as the DICOM term InPlanePhaseEncodingDirection which can have "ROW" or "COL" values. **This parameter is required if a corresponding fieldmap data is present or when using multiple runs with different phase encoding directions (which can be later used for field inhomogeneity correction).**
- `EffectiveEchoSpacing`: The sampling interval also known as the dwell time; required for unwarping distortions using field maps; **expressed in seconds.** See here how to calculate it. **This parameter is required if a corresponding fieldmap data is present.**

[5] http://fsl.fmrib.ox.ac.uk/fsl/fsl4.0/fdt/fdt_dtifit.html

- `EchoTime`: The echo time (TE) for the acquisition, specified in seconds. May be a list of values separated for multi-echo acquisitions. **This parameter is required if a corresponding fieldmap data is present.**
- `TotalReadoutTime`: defined as the time (in seconds) from the center of the first echo to the center of the last echo (see here and here how to calculate it).

Example JSON file:

8.8.3 json example:

```
{
    "PhaseEncodingDirection": "j-",
    "TotalReadoutTime": 0.000095,
}
```

8.9 Fieldmap data

Data acquired to correct for B0 inhomogeneities can come in different forms. The current version of this standard considers four different scenarios. Please note that in all cases fieldmap data can be linked to a specific scan(s) it was acquired for by filling the `IntendedFor` field in the corresponding JSON file. For example:

```
{
   "IntendedFor": "func/sub-01_task-motor_bold.nii.gz"
}
```

The `IntendedFor` field consists of one or more relative paths stripped from the “sub-<label>”. Here’s an example with multiple target scans:

```
{
   "IntendedFor": ["ses-pre/func/sub-01_task-motor_run-01_bold.nii.gz",
                   "ses-post/func/sub-01_task-motor_run-01_bold.nii.gz"]
}
```

The IntendedFor field is optional and in case the fieldmaps do not correspond to any particular scans it does not have to be filled.

Multiple fieldmaps can be stored. In such case the “`_run-01`”, “`_run-02`” should be used.

8.9.1 Case 1: Phase difference image and at least one magnitude image

Template:

```
sub-<participant_label>/
      fmap/
            sub-<label>_phasediff.nii[.gz]

sub-<participant_label>/
      fmap/
```

```
            sub-<label>_phasediff.json

sub-<participant_label>/
      fmap/
            sub-<label>_magnitude1.nii[.gz]
```

(optional)

```
sub-<participant_label>/
      fmap/
            sub-<label>_magnitude2.nii[.gz]
```

This is a common output for build in fieldmap sequence on Siemens scanners. In this particular case the sidecar JSON file has to define the Echo Times of the two phase images used to create the difference image. For example:

```
{
    "EchoTime1": 0.00600,
    "EchoTime2": 0.00746,
    "IntendedFor": "func/sub-01_task-motor_bold.nii.gz"
}
```

8.9.2 Case 2: Two phase images and two magnitude images

Template:

```
sub-<participant_label>/
      fmap/
            sub-<label>_phase1.nii[.gz]

sub-<participant_label>/
      fmap/
            sub-<label>_phase1.json

sub-<participant_label>/
      fmap/
            sub-<label>_phase2.nii[.gz]

sub-<participant_label>/
      fmap/
            sub-<label>_phase2.json

sub-<participant_label>/
      fmap/
            sub-<label>_magnitude1.nii[.gz]
```

```
sub-<participant_label>/
      fmap/
            sub-<label>_magnitude2.nii[.gz]
```

Similar to the case above, but instead of a precomputed phase difference map two separate phase images are presented. The two sidecar JSON file need to specify corresponding EchoTime values. For example:

```
{
    "EchoTime": 0.00746,
    "IntendedFor": "func/sub-01_task-motor_bold.nii.gz"
}
```

8.9.3 Case 3: A single, real fieldmap image (showing the field inhomogeneity in each voxel)

Template:

```
sub-<participant_label>/
      fmap/
            sub-<label>_magnitude.nii[.gz]

sub-<participant_label>/
      fmap/
            sub-<label>_fieldmap.nii[.gz]

sub-<participant_label>/
      fmap/
            sub-<label>_fieldmap.json
```

In some cases (for example GE) the scanner software will output a precomputed fieldmap denoting the B0 inhomogeneities along with a magnitude image used for coregistration. In this case the sidecar JSON file needs to include the units of the fieldmap. The possible options are: “Hz”, “rad/s”, or “Tesla”. For example:

```
{
    "Units": "rad/s",
    "IntendedFor": "func/sub-01_task-motor_bold.nii.gz"
}
```

8.9.4 Case 4: Multiple phase encoded directions (topup)

Template:

```
sub-<participant_label>/
      fmap/
            sub-<label>_dir-<index>_epi.nii[.gz]

sub-<participant_label>/
```

```
fmap/
      sub-<label>_dir-<index>_epi.json
```

This technique combines two or more Spin Echo EPI scans with different phase encoding directions. In such a case, the phase encoding direction is specified in the corresponding JSON file as one of: "i", "j", "k", "i-", "j-, "k-". For these differentially phase encoded sequences, one also needs to specify the Total Readout Time defined as the time (in seconds) from the center of the first echo to the center of the last echo (see here and here how to calculate it). For example

```
{
    "PhaseEncodingDirection": "j-",
    "TotalReadoutTime": 0.000095,
    "IntendedFor": "func/sub-01_task-motor_bold.nii.gz"
}
```

8.10 Scans key file

Template:

```
sub-<participant_label>/
      sub-<participant_label>_scans.tsv
```

Optional: yes

The purpose of this file is to describe timing and other properties of each imaging acquisition sequence (each run .nii[.gz] file) within one session. Each .nii[.gz] file should be described by at most one row. Relative paths to files should be used under a compulsory "filename" header.
If acquisition time is included it should be under "acq_time" header. Datetime should be expressed in the following format `2009-06-15T13:45:30` (year, month, day, hour (24h), minute, second; this is equivalent to the RFC3339 "date-time" format, time zone is always assumed as local time). For anonymization purposes all dates within one subject should be shifted by a randomly chosen (but common across all runs etc.) number of days. This way relative timing would be preserved, but chances of identifying a person based on the date and time of their scan would be decreased. Dates that are shifted for anonymization purposes should be set to a year 1900 or earlier to clearly distinguish them from unmodified data. Shifting dates is recommended, but not required.
Additional fields can include external behavioural measures relevant to the scan. For example vigilance questionnaire score administered after a resting state scan.

8.10.1 Example:

```
filename                                  acq_time
func/sub-control01_task-nback_bold.nii.gz      1877-06-15T13:45:30
```

`func/sub-control01_task-motor_bold.nii.gz      1889-06-15T13:55:33`

8.11 Participant key file

Template:
(single session case)
`participants.tsv`

Optional: Yes

The purpose of this file is to describe properties of participants such as age, handedness, gender etc. In case of single session studies this file has one compulsory column "`participant_id`" followed by a list of optional columns describing participants. Each participant needs to be described by one and only one row.

8.11.1 Single session example:

```
participant_id   age   sex   group
sub-control01    34    M     control
sub-control02    12    F     patient
sub-patient01    33    F     control
```

9 Longitudinal studies with multiple sessions (visits)

Multiple sessions (visits) are encoded by adding an extra layer of directories and file names in the form of `ses-<session_label>`. Session label can consist only of alphanumeric characters [a-zA-Z0-9] and should be consistent across subjects. If numbers are used in session labels we recommend using zero padding (for example `ses-01`, `ses-11` instead of `ses-1`, `ses-11`). This makes results of alphabetical sorting more intuitive. Acquisition time of session can be defined in the sessions file (see below for details).

The extra session layer (at least one `/ses-<session_label>` subfolder) should be added for all subjects if at least one subject in the dataset has more than one session. Skipping the session layer for only some subjects in the dataset is not allowed.

- **`sub-control01`**
    - **`ses-predrug`**
        - **`anat`**
            - `sub-control01_ses-predrug_T1w.nii.gz`
            - `sub-control01_ses-predrug_T1w.json`
            - `sub-control01_ses-predrug_T2w.nii.gz`
            - `sub-control01_ses-predrug_T2w.json`
        - **`func`**
            - `sub-control01_ses-predrug_task-nback_bold.nii.gz`
            - `sub-control01_ses-predrug_task-nback_bold.json`
            - `sub-control01_ses-predrug_task-nback_events.tsv`

            - sub-control01_ses-predrug_task-nback_cont-physio.tsv.gz
            - sub-control01_ses-predrug_task-nback_cont-physio.json
            - sub-control01_ses-predrug_task-nback_sbref.nii.gz
        - **dwi**
            - sub-control01_ses-predrug_dwi.nii.gz
            - sub-control01_ses-predrug_dwi.bval
            - sub-control01_ses-predrug_dwi.bvec
        - **fmap**
            - sub-control01_ses-predrug_phasediff.nii.gz
            - sub-control01_ses-predrug_phasediff.json
            - sub-control01_ses-predrug_magnitude1.nii.gz
        - sub-control01_ses-predrug_scans.tsv
    - **ses-postdrug**
        - **func**
            - sub-control01_ses-postdrug_task-nback_bold.nii.gz
            - sub-control01_ses-postdrug_task-nback_bold.json
            - sub-control01_ses-postdrug_task-nback_events.tsv
            - sub-control01_ses-postdrug_task-nback_cont-physio.tsv.gz
            - sub-control01_ses-postdrug_task-nback_cont-physio.json
            - sub-control01_ses-postdrug_task-nback_sbref.nii.gz
        - **fmap**
            - sub-control01_ses-postdrug_phasediff.nii.gz
            - sub-control01_ses-postdrug_phasediff.json
            - sub-control01_ses-postdrug_magnitude1.nii.gz
        - sub-control01_ses-postdrug_scans.tsv
    - sub-control01_sessions.tsv
- participants.tsv
- dataset_description.json
- README
- CHANGES

9.1 Sessions file

Template:

```
sub-<participant_label>/
      sub-<participant_label>_sessions.tsv
```

Optional: Yes

In case of multiple sessions there is an option of adding an additional participant key files describing variables changing between sessions. In such case one file per participant should be added. These files need to include compulsory "`session_id`" column and describe each session by one and only one row. Column names in per participant key files has to be different from group level participant key column names.

9.1.1 Multiple sessions example:

session_id	acq_time	systolic_blood_pressure
ses-predrug	2009-06-15T13:45:30	120
ses-postdrug	2009-06-16T13:45:30	100
ses-followup	2009-06-17T13:45:30	110

12 Appendix I: Licenses

This section lists a number of common licenses for datasets and defines suggested abbreviations for use in the dataset metadata specifications

PD	Public Domain	No license required for any purpose; the work is not subject to copyright in any jurisdiction.
PDDL	Open Data Commons Public Domain Dedication and License	License to assign public domain like permissions without giving up the copyright: http://opendatacommons.org/licenses/pddl/
CC0	Creative Commons Zero 1.0 Universal.	Use this if you are a holder of copyright or database rights, and you wish to waive all your interests in your work worldwide: https://creativecommons.org/about/cc0
